# Emergency Partington-Rochelle Procedure for Hemorrhagic Peritonitis Due to Stent Migration Following Endoscopic Ultrasonography-Guided Pancreatic Duct Drainage

**DOI:** 10.7759/cureus.83384

**Published:** 2025-05-03

**Authors:** Takehiko Hanaki, Hirotaka Tanabe, Teruhisa Sakamoto, Masaru Ueki, Yoshiyuki Fujiwara

**Affiliations:** 1 Department of Gastrointestinal and Pediatric Surgery, Tottori University Faculty of Medicine, Yonago, JPN; 2 Department of Medical Education, Tottori University Faculty of Medicine, Yonago, JPN

**Keywords:** emergency partington-rochelle procedure, endoscopic ultrasonography-guided pancreatic duct drainage, general surgrery, hemorrhagic peritonitis, stent migration

## Abstract

Endoscopic ultrasonography-guided pancreatic duct drainage (EUS-PD) has become a valuable alternative to conventional retrograde approaches in treating chronic pancreatitis, particularly in cases where transpapillary access is technically unfeasible. Although generally considered safe and effective, EUS-PD can lead to rare but serious complications, such as stent migration, hemorrhage, and peritonitis. The surgical management of these adverse events remains inadequately documented in the clinical literature. We present the case of a 72-year-old man with a long-standing history of alcohol-related chronic pancreatitis and pancreatic diabetes who developed severe epigastric pain due to multiple intraductal pancreatic stones. Several attempts at endoscopic retrograde pancreatic lithotomy failed due to anatomical difficulties. EUS-PD was subsequently performed, and a covered self-expandable metal stent was placed transgastrically into the main pancreatic duct (MPD). Immediately after deployment, bleeding into the gastric lumen was observed. Contrast-enhanced computed tomography revealed stent migration from the MPD with active intraabdominal hemorrhage. The patient rapidly developed signs of peritonitis and hemodynamic instability, prompting an emergency laparotomy. Intraoperatively, the stent was found to have perforated the lesser and posterior gastric walls and was partially embedded in the pancreatic parenchyma. After removal of the stent, the gastric perforation was repaired, and hemostasis was achieved. A longitudinal pancreaticojejunostomy (Partington-Rochelle procedure) was performed to manage the injured and dilated pancreatic duct. The postoperative course was uneventful, with complete resolution of abdominal symptoms and no development of a pancreatic fistula. This case highlights the need for heightened awareness of potentially life-threatening EUS-PD-related complications. It also underscores the importance of timely surgical intervention and demonstrates that the Partington-Rochelle procedure can be a practical and effective option in emergency settings for managing ductal disruption caused by stent migration.

## Introduction

Endoscopic ultrasonography-guided pancreatic duct drainage (EUS-PD) has emerged as a valuable therapeutic option for patients with obstructive pancreatitis in whom conventional endoscopic retrograde pancreatic drainage is unsuccessful or unfeasible [1,2]. This technique enables direct access to the main pancreatic duct (MPD) via a transgastric or transduodenal route under ultrasonography guidance and has shown high technical and clinical success rates, particularly in chronic pancreatitis with ductal obstruction [1-3]. While endoscopic intervention remains the standard initial treatment for painful chronic pancreatitis in Japan [4], it should be noted that international guidelines and recent studies, including the Evaluation Study of Congestive Heart Failure and Pulmonary Artery Catheterization Effectiveness (ESCAPE) trial [5] and the International Consensus Guidelines [6], recommend early surgical intervention to achieve better pain control and quality of life. Therefore, although early surgery is now considered the first-line treatment strategy in many Western countries, endoscopic interventions, including endoscopic retrograde procedures and EUS-guided approaches, have remained the standard initial therapy in Japan, in accordance with the Japanese Clinical Practice Guidelines for Chronic Pancreatitis [4]. The management strategy described in this case reflects the regional standard practice at the time of treatment.

As its adoption escalates across specialized centers, EUS-PD has been linked to a range of procedure-related complications [7,8], including bleeding, infection, pancreatitis, perforation, pancreatic fistula, and stent-related events, including mal-deployment, occlusion, or migration [7-9]. Among these events, intraperitoneal stent migration is considered extremely rare. To the best of our knowledge, although it has been mentioned in the summary of EUS-PD complications [8], no detailed individual case reports describing its clinical course and surgical management have been published to date. This complication may lead to severe outcomes, including hemorrhage and peritoneal contamination with pancreatic juice, requiring urgent surgical intervention.

Herein, we present a unique case of hemorrhagic peritonitis due to stent migration following EUS-PD, which was successfully managed with an emergency longitudinal pancreaticojejunostomy (Partington-Rochelle procedure) [10]. This case underscores the significance of surgical awareness of EUS-PD-related complications and offers insights into operative strategies for the simultaneous management of acute and chronic pancreatic conditions, even in emergent settings.

## Case presentation

**Figure 1:**
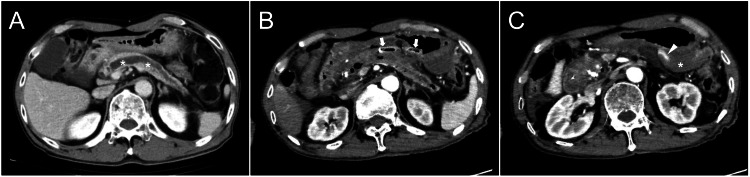
Contrast-enhanced axial computed tomography images obtained before the procedure and after confirmation of bleeding following stent insertion (A) Preprocedural image showing a dilated MPD (*). (B) The stent (arrows) penetrating the gastric wall has migrated from the MPD. Notably, gas density is observed within the MPD, indicating that the stent had indeed been inserted into the duct before migration occurred. (C) Hematoma formation (*) within the omental bursa and contrast medium extravasation (arrowhead) are confirmed. MPD: main pancreatic duct

A 72-year-old man with a long-standing history of alcohol-related chronic pancreatitis was referred to our department for surgical evaluation and treatment of acute complications following an EUS-PD procedure. He had been experiencing recurrent epigastric pain for over a decade, which was attributed to pancreatic calculi in the head lesion and chronic ductal obstruction. The pain was chronically present and perceived as persistent epigastric discomfort associated with chronic pancreatitis; however, it was not notably exacerbated by food intake. Previous computed tomography had consistently revealed multiple pancreatic calculi within a significantly dilated MPD, predominantly affecting the entire pancreas (Figure 1A).

His comorbidities comprised a prior cerebral infarction, for which he was receiving cilostazol, and pancreatic diabetes mellitus, with suboptimal glycemic control (glycated hemoglobin, 7.4%).

In the previous months, our institution’s biliary-pancreatic endoscopists had attempted several endoscopic retrograde pancreatography sessions to remove the pancreatic calculi in the pancreatic head and decompress the MPD. Despite repeated efforts, stone extraction via the transpapillary route remained technically difficult, and a plastic drainage catheter was temporarily placed within the MPD for symptomatic relief. On the day of referral to our department, a further attempt at endoscopic lithotripsy was again unsuccessful owing to difficulty in accessing the pancreatic duct through the papilla. MPD cannulation could not be achieved, even after extended manipulation.

Considering the persistent ductal obstruction and the patient’s persistent epigastric pain, the biliary-pancreatic endoscopist team decided to proceed with EUS-PD, targeting the MPD at the level of the pancreatic body. Under endosonographic guidance, the MPD was successfully punctured, and a guidewire was advanced into the MPD. However, gastric luminal bleeding through the placed covered self-expandable metallic stent (6-mm diameter; Hanarostent Benefit, M.I.Tech Co., Ltd., Seoul, South Korea) developed following placement of the stent that should have been inserted into the MPD. The endoscopic procedure was terminated, and contrast-enhanced computed tomography was performed, revealing stent migration from the MPD with associated active intraabdominal hemorrhage (Figures 1B-1C). A surgical consultation was immediately requested.

The patient exhibited signs of developing peritonitis with hemodynamic instability. Based on the radiological and clinical findings, an emergency surgical intervention was required to control the hemorrhage, manage the pancreatic injury, and prevent further complications arising from intraabdominal pancreatic juice leakage.

**Figure 2:**
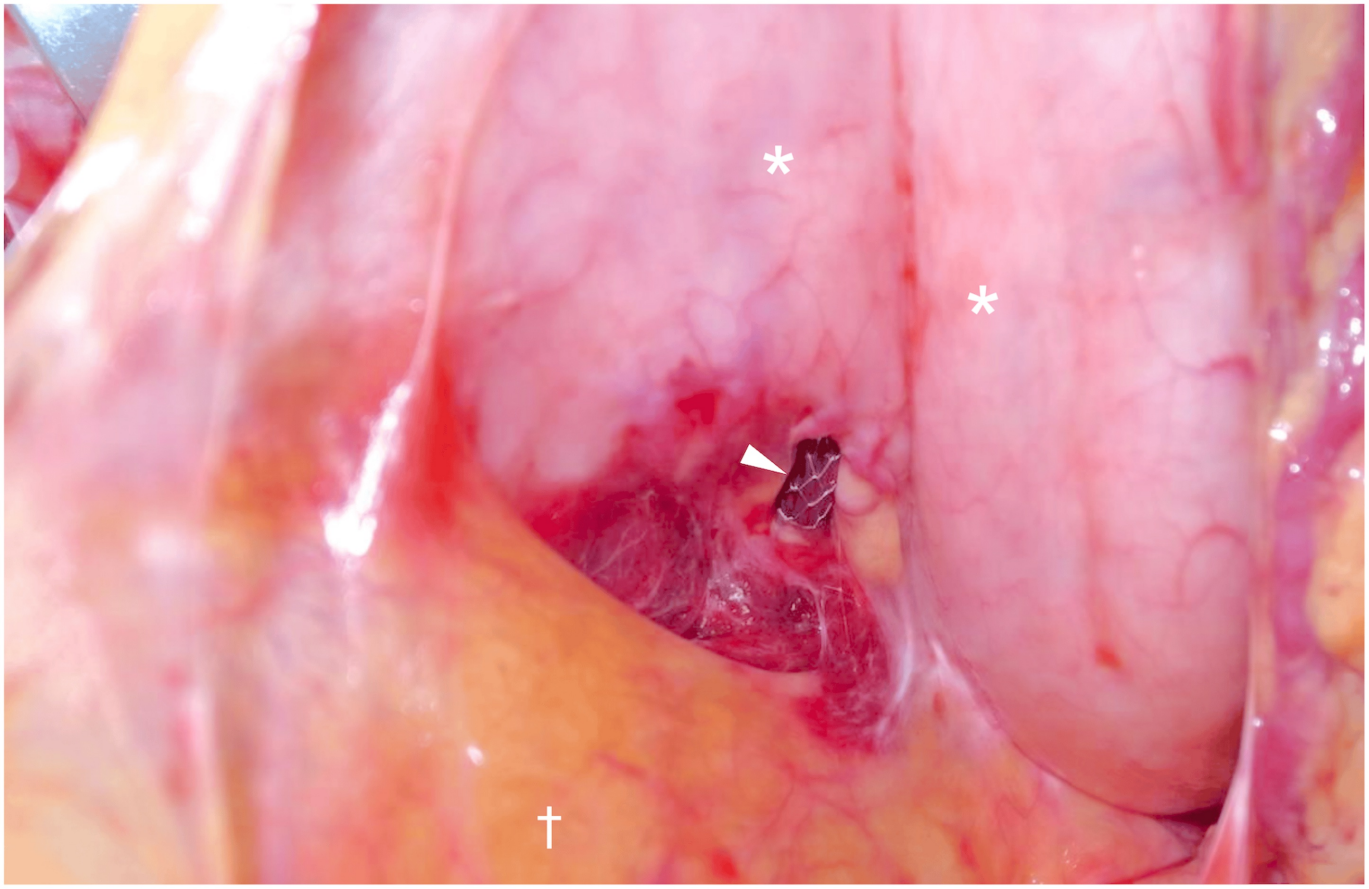
Intraoperative findings in the omental bursa An intraoperative image revealing the migrated metallic stent (arrowhead) penetrating the lesser and posterior gastric walls (*) and partially embedded in the pancreatic parenchyma. To expose the omental bursa, the transverse mesocolon (†) is retracted caudally.

An emergency laparotomy was performed. Upon accessing the abdominal cavity, a moderate amount of bloody ascites and hematoma was observed, particularly within the omental bursa. The stent was identified as protruding through the lesser and posterior gastric walls, partially embedded in the pancreatic parenchyma (Figure 2).

**Figure 3:**
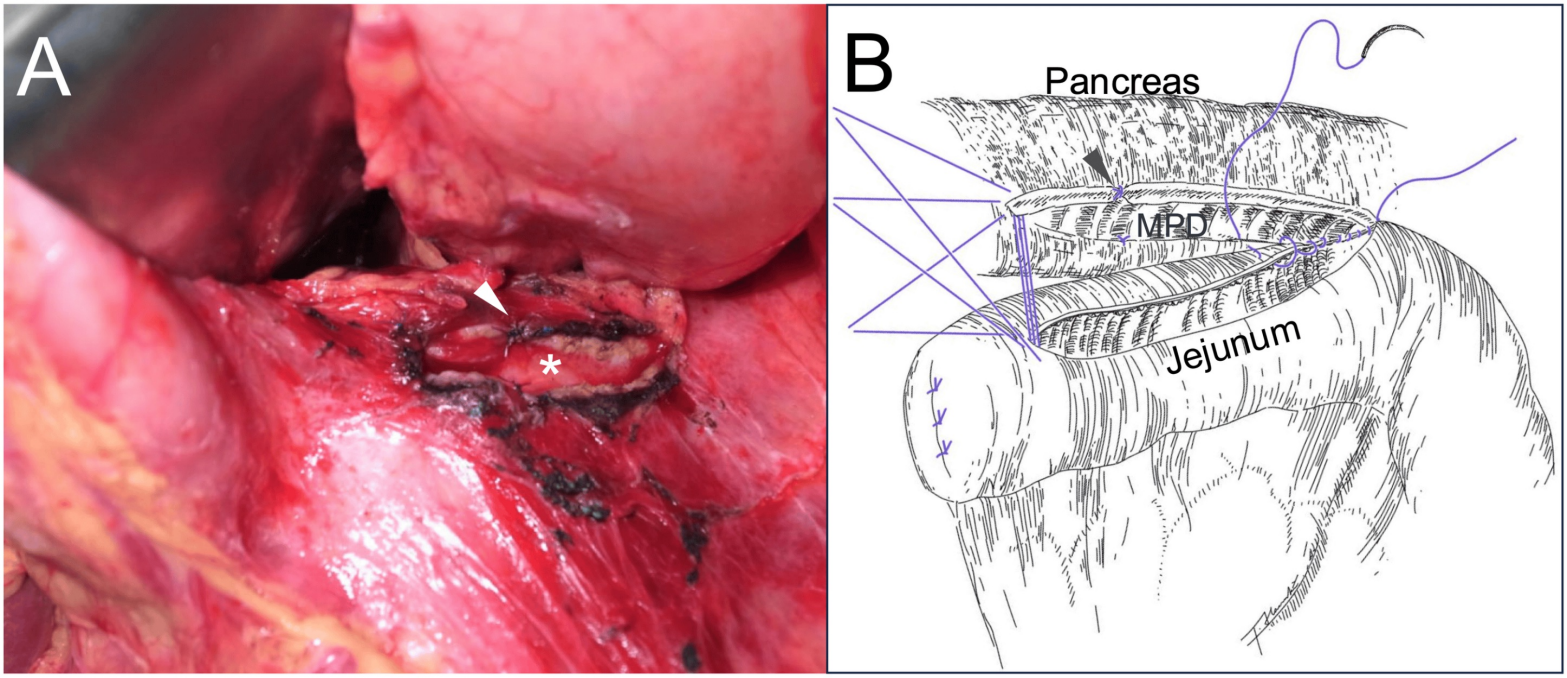
Image of the incised MPD and reconstruction scheme (A) For pancreaticojejunostomy, the puncture site of the pancreas is incised in the longitudinal direction to expose approximately 5 cm of the MPD (*). An arrowhead indicates a hemostatic suture placed on the pancreatic parenchyma at the site of stent penetration. The longitudinal pancreatic ductotomy (*) is visible. (B) A diagram of the pancreaticojejunostomy procedure. Several 4-0 absorbable sutures are placed at the long end, and the anastomosis is completed using a continuous suture. The site of hemostatic suture placement corresponding to the stent penetration site is also marked with an arrowhead. MPD: main pancreatic duct Credit: Illustration created by Takehiko Hanaki

It was carefully retrieved. Using a two-layer closure technique, the gastric iatrogenic penetration site was repaired. Active bleeding from the pancreatic parenchyma was controlled using direct suture. Pancreatic body exploration revealed that the MPD was exposed at the site of parenchymal disruption caused by the stent insertion and migration. The ductotomy was subsequently extended both proximally and distally from this disrupted segment, along the long axis of the pancreas, in preparation for reconstruction (Figure 3A).

Considering the substantial calculus load in the pancreatic head and the patient’s unstable preoperative condition, further pancreatic head region exploration was deferred. Reconstruction was performed by creating a side-to-side pancreaticojejunostomy using a Roux-en-Y limb (Partington-Rochelle procedure, Figure 3B). To facilitate postoperative monitoring, two silicone drains, one superior and one inferior to the anastomotic site, were placed.

The total operative time was 191 minutes, and the estimated blood loss was 250 mL, including preoperative intra-abdominal bleeding. The patient was transferred to the intensive care unit for close observation. No evidence of postoperative pancreatic fistula was observed. On postoperative day (POD) 3, the drains were removed, and cilostazol therapy was safely resumed on the same day. On POD 4, oral intake was recommenced. On POD 8, the patient was transferred to the internal medicine department for further diabetes management.

Preoperatively, the patient reported severe epigastric pain, with a numerical rating scale (NRS) score of 8-9. Postoperatively, his pain completely resolved, with a reported NRS score of 0. Throughout the postoperative period, he remained hemodynamically stable and did not require a blood transfusion. At the latest follow-up, 52 months after surgery, there has been no recurrence of symptoms related to chronic pancreatitis.

## Discussion

EUS-PD has become an essential second-line intervention for patients with obstructive chronic pancreatitis, especially when endoscopic transpupillary retrograde approaches are unsuccessful owing to anatomical limitations or ductal obstructions [1,2,7,11]. Recent advancements in interventional endoscopy have enabled increasing utilization of EUS-PD, demonstrating promising outcomes with high technical success rates of 75-100% in experienced centers [8,12].

Despite its growing adoption, EUS-PD poses inherent risks. Recent reviews published in the 2020s have reported that EUS-PD is associated with complication rates ranging from 14% to 40%, depending on patient selection, technical approach, and operator experience [7]. These complications encompass pancreatitis, perforation, bleeding, infection, and stent migration [7,8]. Among these, intraperitoneal stent migration resulting in hemorrhage and peritonitis is extremely rare, and few detailed reports exist in the literature describing its surgical management [8].

In the present case, the stent penetrated the gastric wall, and the pancreatic parenchyma migrated from the MPD, resulting in hemorrhagic peritonitis and pancreatic juice leakage into the abdominal cavity. Although interventional radiology might effectively control the bleeding, the ongoing peritoneal cavity contamination by pancreatic juice and the stent penetrating the gastric wall necessitated surgical intervention. Of note, isolated gastrointestinal perforations may occasionally be conservatively or endoscopically managed, especially when the perforation is contained and exhibits no systemic signs. However, in this instance, transmural penetration into the pancreas and pancreatic and gastric juice leakage rendered nonoperative management inappropriate.

Emergency surgical intervention following EUS-PD-related complications has been sparsely addressed in the literature [8], with most references appearing in tabulated summaries of case series rather than in dedicated case reports. Consequently, the optimal surgical approach remains unclear and relies on expert judgment. The following crucial considerations must be weighed during surgical decision-making: (1) the extent of visceral injury, (2) the necessity for debridement or infectious source control, (3) the integrity of the MPD, and (4) whether to perform a decompressive drainage procedure. In our patient, the injury involved the gastric wall and pancreas, requiring the repair of both structures. Furthermore, the disrupted MPD was associated with upstream dilatation and stone impaction in the pancreatic head. Consequently, decompressive drainage was indicated to prevent further leakage and facilitate healing. The Partington-Rochelle procedure was employed, focusing on dilated body-to-tail segment decompression and controlling contaminations while preserving the pancreatic head to shorten the operative time and avoid unnecessary manipulation. Intraoperatively, the ductal disruption caused by the migrated stent measured only a few millimeters in diameter, which was too small to allow for a safe and tension-free anastomosis. Therefore, to enlarge the anastomotic surface and ensure a secure pancreaticojejunostomy, we extended the ductotomy longitudinally along the dilated MPD. This approach also served to decompress the obstructed upstream duct effectively. Based on these considerations, a longitudinal pancreaticojejunostomy was selected as the optimal surgical strategy for both emergent stabilization and long-term pancreatic ductal drainage.

The Partington-Rochelle procedure, although widely accepted for longitudinal decompression in patients with chronic pancreatitis, is frequently indicated for diffuse ductal dilatation or widespread intraductal calculi [13] and is generally performed in an elective setting. In cases such as ours, where pathology is localized and time is critical, a limited drainage strategy can provide a more effective decompression while reducing surgical morbidity than other pancreaticojejunostomy procedures.

The Frey procedure, which comprises coring out the anterior aspect of the pancreatic head along with longitudinal pancreaticojejunostomy [14], provides the benefit of removing impacted calculi within the pancreatic head and improving long-term ductal decompression. However, it is also associated with a longer operative time and greater surgical complexity than the Partington-Rochelle procedure. In patients with critical illness or emergency settings, these disadvantages may increase the risk of morbidity. In contrast, the Partington-Rochelle procedure provides a technically more straightforward and faster means of achieving adequate pancreatic body and tail decompression and controlling contaminations in EUS-PD complications.

In this case, controlling iatrogenic injury to the pancreas and surrounding viscera while ensuring adequate ductal drainage constitutes the immediate surgical priority. The Partington-Rochelle procedure was both necessary and sufficient considering the localization of the injury and the patient’s unstable condition. Furthermore, we considered that deferring definitive ductal drainage to a secondary operation would have increased surgical difficulty due to postoperative adhesions and posed additional morbidity risks. As the MPD was already dilated and accessible, we judged that completing definitive decompression during the initial emergency surgery would optimize the patient’s clinical outcome and avoid the need for a second surgical intervention. Thus, the decision to perform the Partington-Rochelle procedure at the time of the emergency operation was both justified and appropriate. It successfully resolved the pancreatic juice leakage and provided complete postoperative relief of the patient’s chronic pain. Additional resection or head coring as required in the Frey procedure was considered unnecessary and potentially excessive.

Moreover, this case emphasizes a crucial consideration for general surgeons. As EUS-PD becomes more widely performed by interventional endoscopists, including at centers without immediate hepatopancreatobiliary surgical coverage, complications requiring urgent surgical intervention may unexpectedly arise. Therefore, general surgeons, particularly those in acute care or rural settings, should maintain familiarity with pancreatic ductal drainage procedures, including longitudinal pancreaticojejunostomy. To optimize outcomes in these complex scenarios, early collaboration between the surgical and endoscopic teams remains critical.

Alternatively, a two-phase approach could have been considered, consisting of initial emergency control of hemorrhage and pancreatic leakage, followed by elective definitive ductal drainage after stabilization. This strategy might offer advantages such as reduced operative time during the acute phase and more controlled planning for ductal surgery. However, in this case, the significant dilation of the MPD, the accessibility of the injured site, and the risks associated with reoperation in a contaminated field led us to favor definitive one-stage decompression during emergency surgery.

## Conclusions

This case underscores the significance of vigilance for rare but grave complications associated with EUS-guided pancreatic duct drainage, particularly transmural stent migration leading to hemorrhagic peritonitis. In such cases, expeditious diagnosis and prompt surgical intervention are of the essence. In the presence of visceral perforation and active leakage of pancreatic or gastric contents, non-operative approaches may be inadequate, and definitive surgical management is required to stabilize the patient and prevent further deterioration.

The successful use of the Partington-Rochelle procedure in this emergency context demonstrates its practical value beyond the elective setting, providing effective pancreatic duct decompression and contamination control. As EUS-PD becomes more widespread, general surgeons must be equipped with the knowledge and skills to manage its complications, especially in acute or resource-limited settings. The significance of close interdisciplinary collaboration in ensuring safe and effective outcomes in complex clinical scenarios cannot be overstated. In the present case, initial endoscopic interventions, including successful papillary cannulation, were attempted in accordance with standard clinical practice. However, due to persistent ductal obstruction by pancreatic stones and subsequent complications after EUS-PD, urgent surgical intervention became necessary. This case highlights the importance of individualized treatment planning based on evolving clinical conditions. Thus, in carefully selected cases, definitive decompression during emergency surgery may provide a favorable clinical outcome and avoid the need for secondary interventions.
